# Supplementary material for: A miniaturized bionic ocean-battery mimicking the structure of marine microbial ecosystems
Source: Nat Commun. 2022 Sep 24;13:5608. doi: 10.1038/s41467-022-33358-x (PMC9509365; doi:10.1038/s41467-022-33358-x)
Supplement: Supplementary file 1 — Supplementary Information [file 41467_2022_33358_MOESM1_ESM.pdf]

A miniaturized bionic ocean-battery mimicking the structure of marine microbial ecosystems

Zhu *et al.*

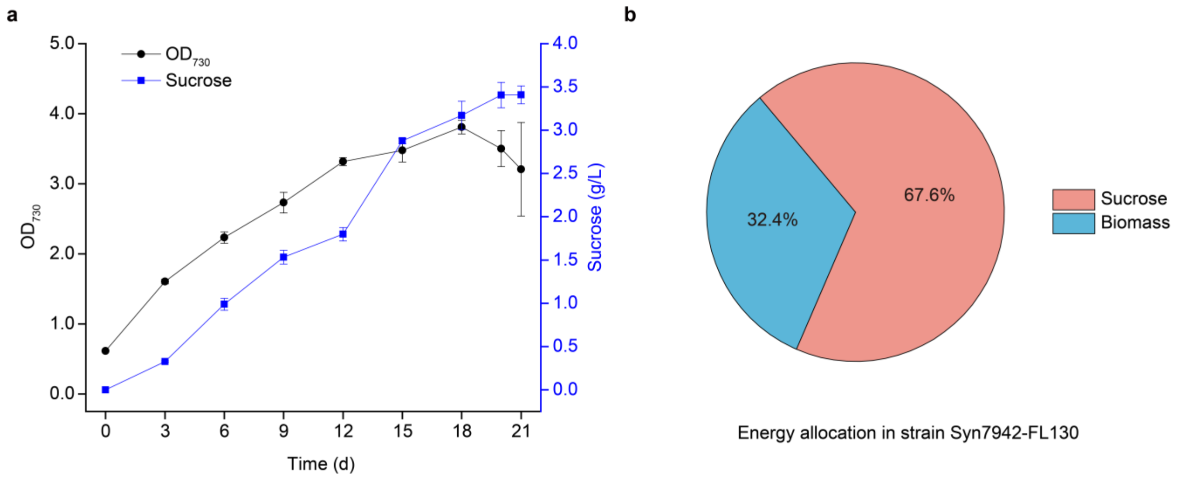

Supplementary Fig. 1. Photosynthetic sucrose production by cyanobacterial strain Syn7942-FL130. a, The growth and sucrose production of the strain in MBG11-S medium. Data are presented as mean values ± SD from n = 3 independent experiments. **b**, Energy allocation in the strain cultivated for 15 d, calculated based on the heat of combustion. Source data are provided as a Source Data file.

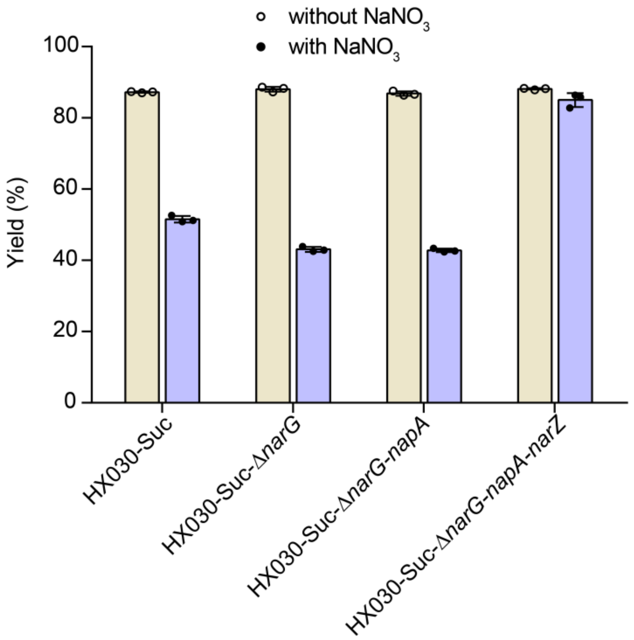

Supplementary Fig. 2. The yield of sucrose to D-lactate by the engineered strains of *E. coli*. To enable strain *E. coli* HX030-Suc to produce D-lactate from sucrose in nitrate-containing medium, the genes encoding three nitrate reductases (NarG, NapA, NarZ) were knocked out successively. The fermentation was conducted in MBG11-S medium with or without $NaNO_3$, supplemented with 0.5 $g \cdot L^{-1}$ tryptone and 0.25 $g \cdot L^{-1}$ yeast extract as nitrogen source. The carbon source was 3 $g \cdot L^{-1}$ sucrose. Data are presented as mean values ± SD from $n = 3$ independent experiments. Source data are provided as a Source Data file.

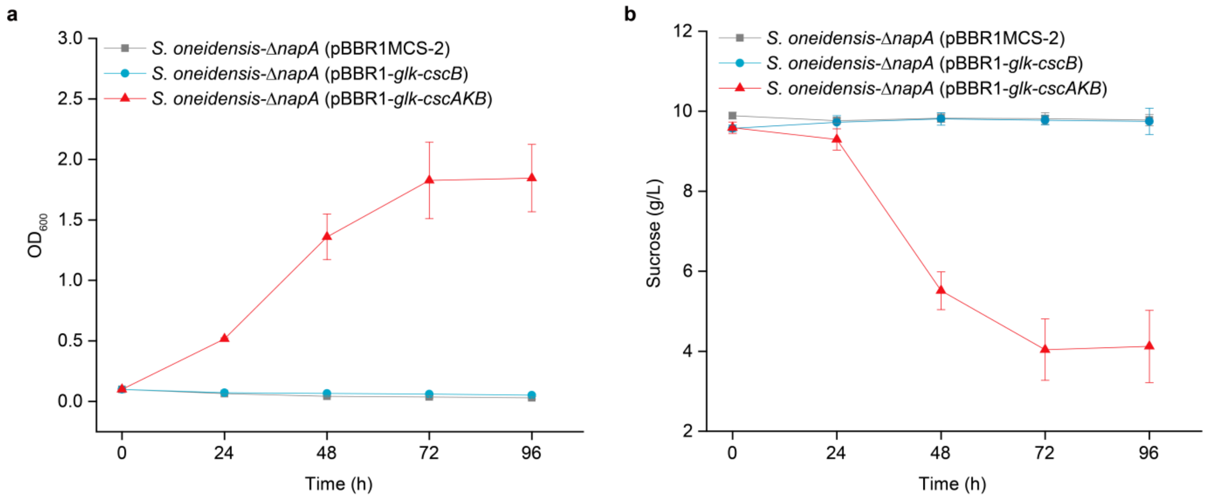

Supplementary Fig. 3. Sucrose utilization by the engineered strains of *S. oneidensis*. a, The growth of three engineered strains harboring different plasmids. Only the gene cassette of *glk-cscAKB* is able to remedy gene deficiency of sucrose catabolism in *S. oneidensis*. **b**, The sucrose consumption of three engineered strains. The growth medium was MBG11-S supplemented with 1.0 g·L^{-1} NH_4Cl as nitrogen source. The carbon source was 10 g·L^{-1} sucrose. Data are presented as mean values ± SD from *n* = 3 independent experiments. Source data are provided as a Source Data file.

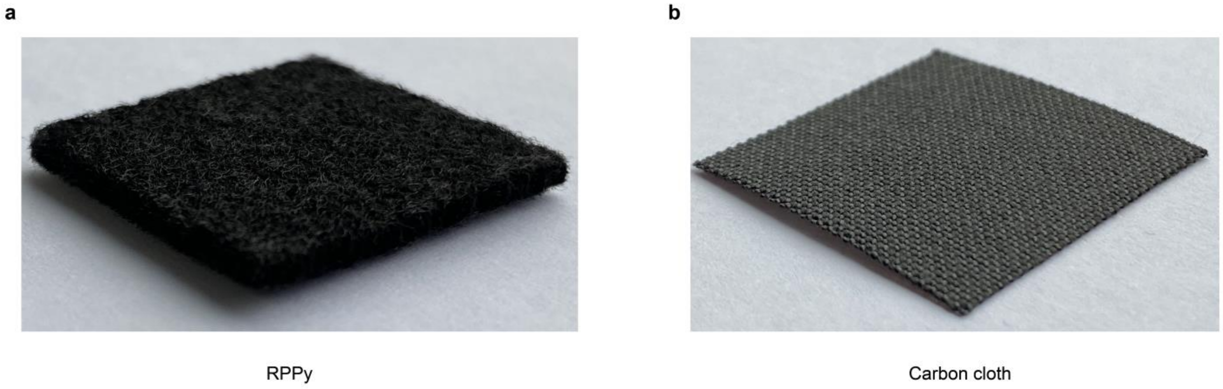

Supplementary Fig. 4. The actual photographs of two types of porous electrode used in this study. a, RPPy electrode. **b**, Carbon cloth. The size was 2.5×2.5 cm. The thickness of RPPy was larger than that of the carbon cloth. Source data are provided as a Source Data file.

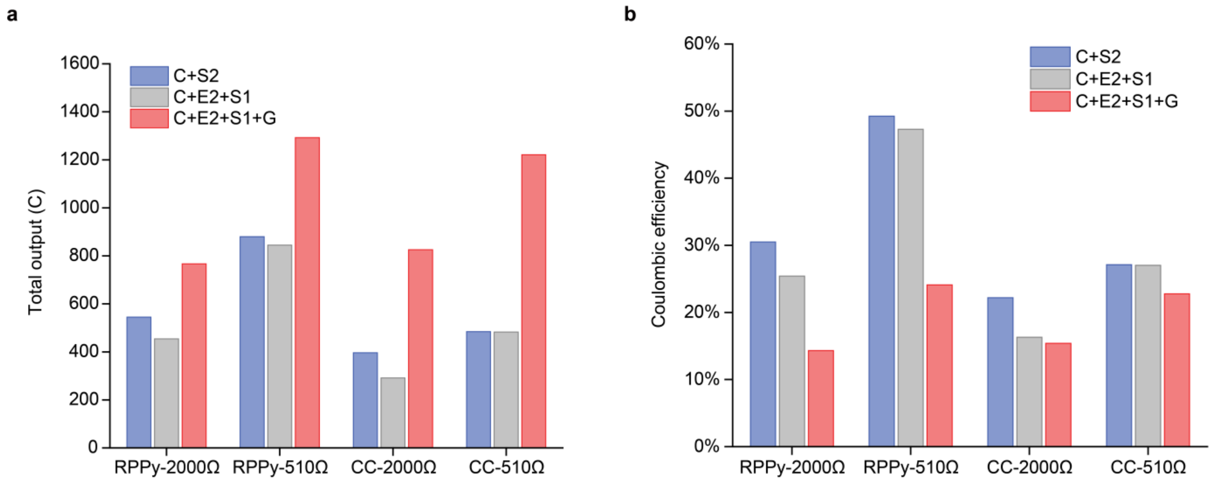

Supplementary Fig. 5. Coulombic efficiency of three synthetic microbial communities. a, The amount of coulombs generated by three synthetic microbial communities. The values are derived from the integral areas of the current-time curves in Fig. 2d-g. **b**, The coulombic efficiency of three synthetic microbial communities. The calculation for C+S2 and C+E2+S1 systems was based on the maximum coulombs released by the oxidation of sucrose to acetate, while the calculation for C+E2+S1+G system was based on the maximum coulomb released from sucrose to CO_2. Abbreviation: CC: carbon cloth. Source data are provided as a Source Data file.

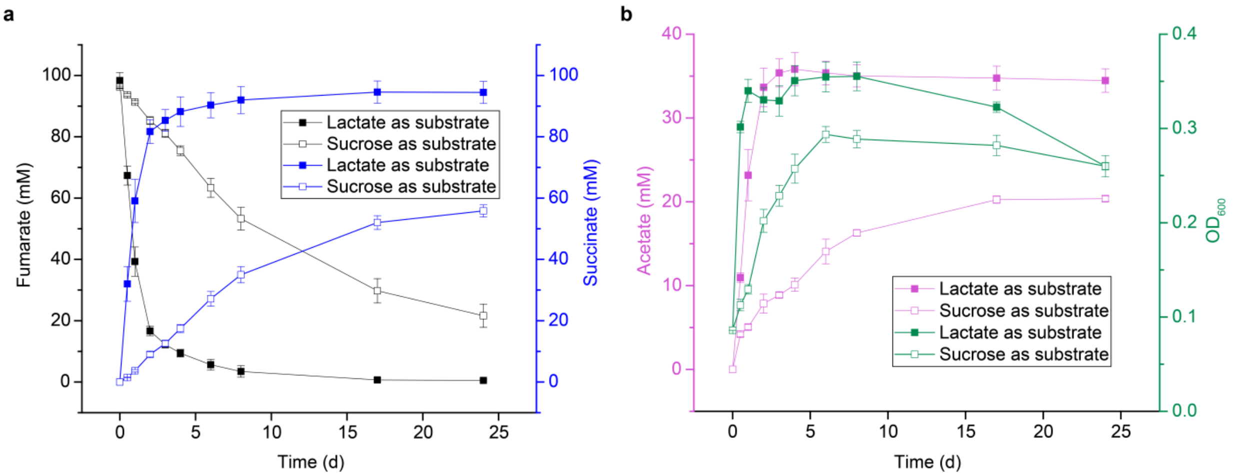

Supplementary Fig. 6. Anaerobic oxidation of two substrates by the engineered strain of *S. oneidensis*. **a**, Fumarate reduction and succinate production of the engineered strain S2. **b**, The growth and acetate production of the engineered strain S2. The medium was MBG11-S supplemented with 0.25 g·L^{-1} tryptone, 0.125 g·L^{-1} yeast extract, 100 mM sodium fumarate as electron acceptor, and 35 mM sodium lactate or 3.0 g·L^{-1} sucrose as substrate. The experiments were conducted anaerobically in sealed bottles. Data are presented as mean values ± SD from n = 3 independent experiments. Source data are provided as a Source Data file.

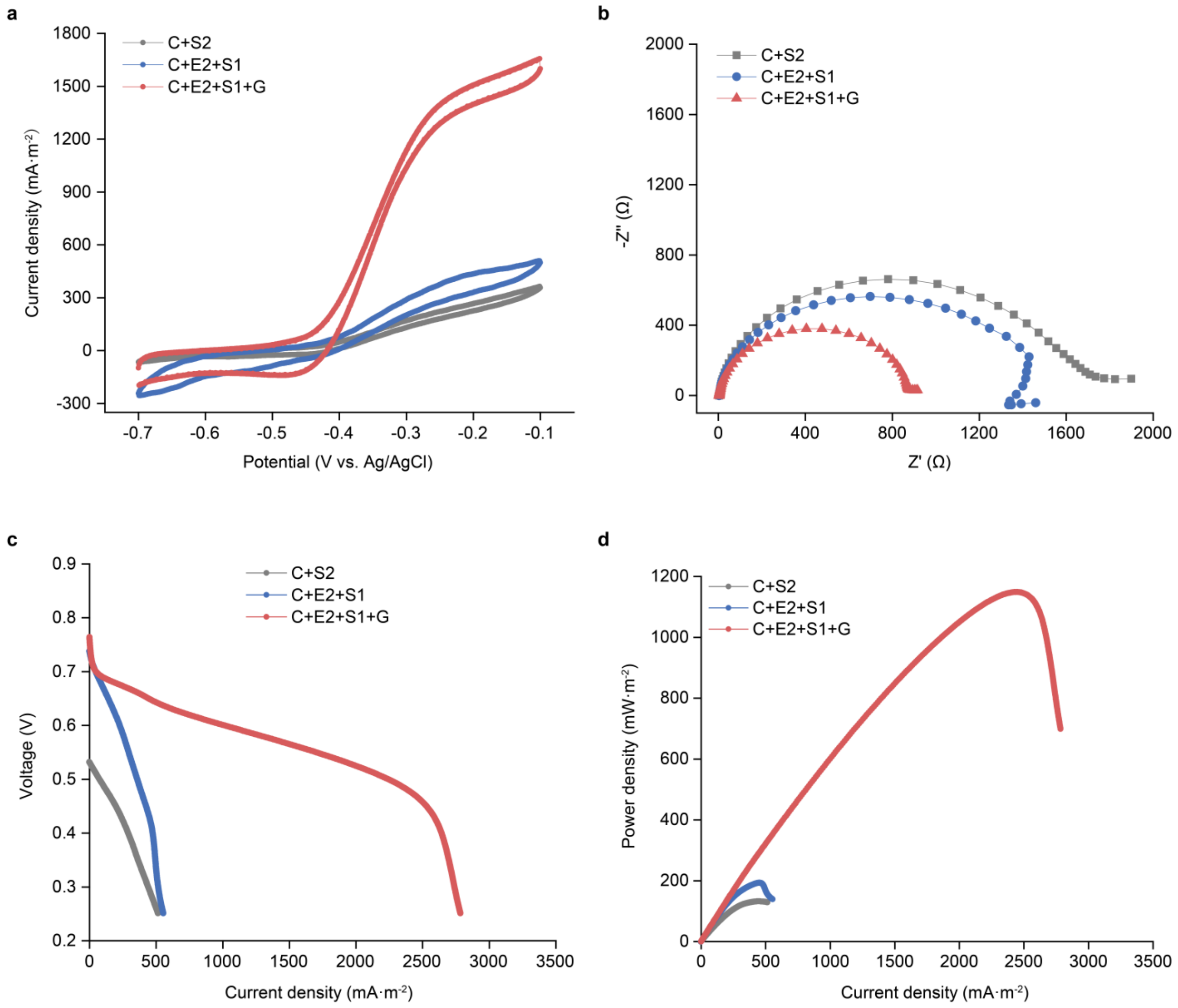

Supplementary Fig. 7. Electrochemical characterization of three synthetic microbial communities attached to the carbon cloth. a, CV curves at a scan rate of 1 mV·s^{-1}. **b**, Nyquist plots of the anode in a frequency range of 100 kHz to 1 mHz. **c**, Polarization curves obtained from LSV. **d**, Power curves derived from polarization curves. The current/power densities were normalized to the geometrical area of anode. The external resistance for anode acclimation before the measurement was 510 Ω. Source data are provided as a Source Data file.

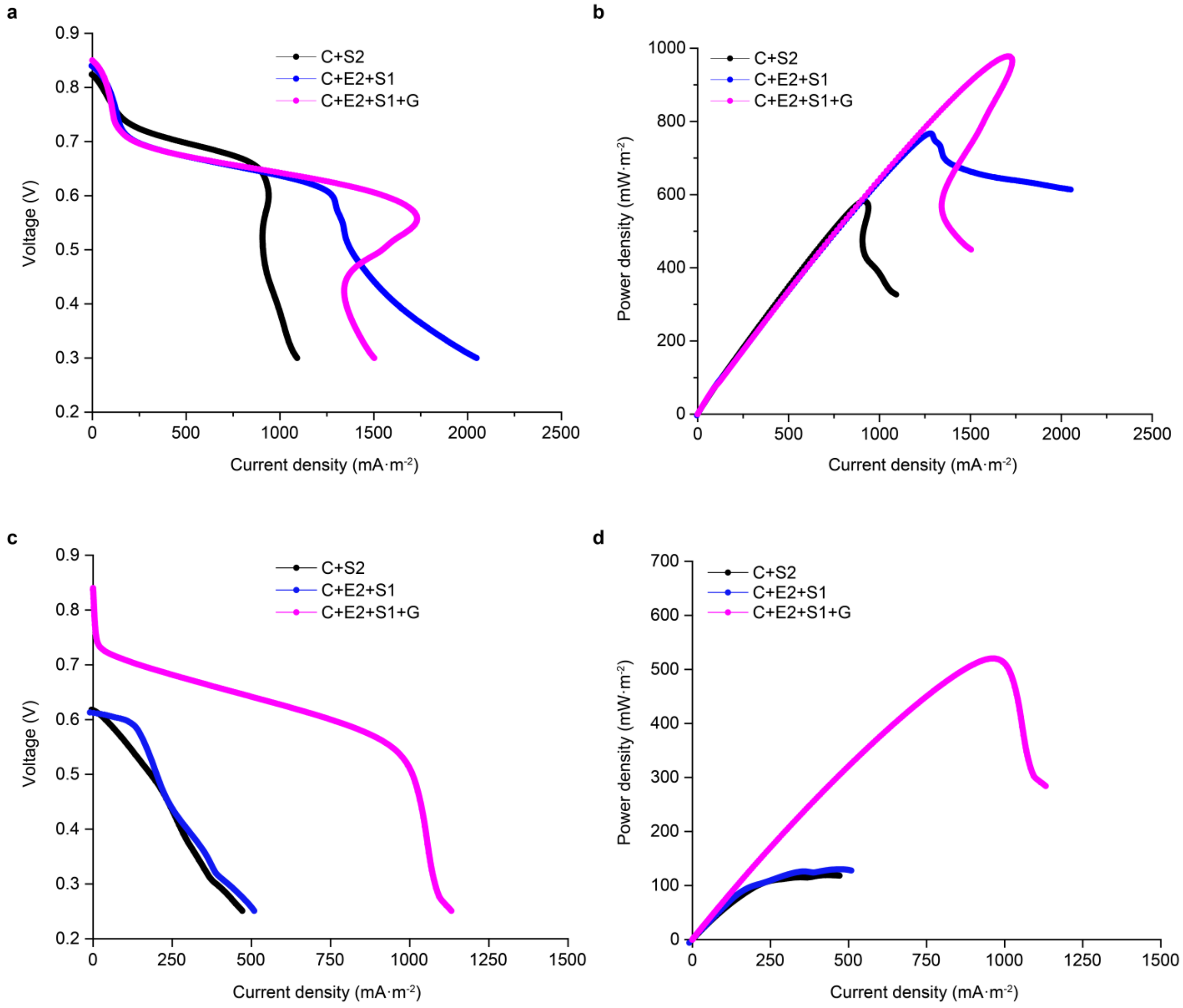

Supplementary Fig. 8. Polarization curves and power curves of the synthetic microbial communities. a,**b**, Polarization curves (**a**) and power curves (**b**) of three synthetic microbial communities with RPPy as electrode. **c**,**d**, Polarization curves (**c**) and power curves (**d**) of three synthetic microbial communities with carbon cloth as electrode. The current/power densities were normalized to the geometrical area of anode. The external resistance for anode acclimation before the measurement was 2000 Ω. Source data are provided as a Source Data file.

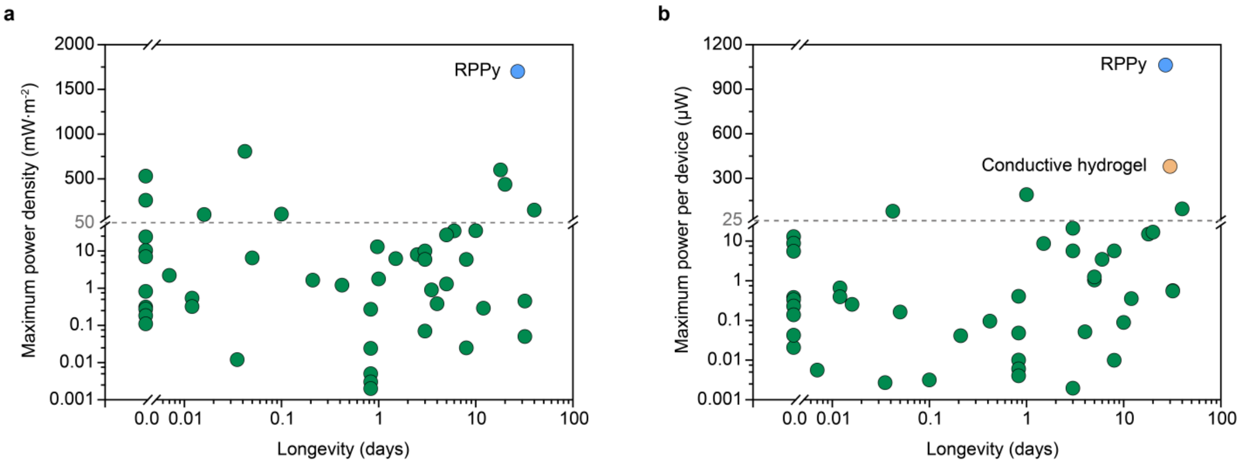

Supplementary Fig. 9. The performance comparison of four-species microbial community with previously demonstrated bio-solar cells. a, Maximum power densities normalized to the geometric area of the anode. **b**, Maximum power output per device. The green circles correspond to the performance of previously demonstrated bio-solar cells (Supplementary Data 1). The light blue and light brown circles correspond to the RPPy-supported four-species system and the conductive hydrogel-based miniaturized bionic ocean-battery, respectively. Source data are provided as a Source Data file.

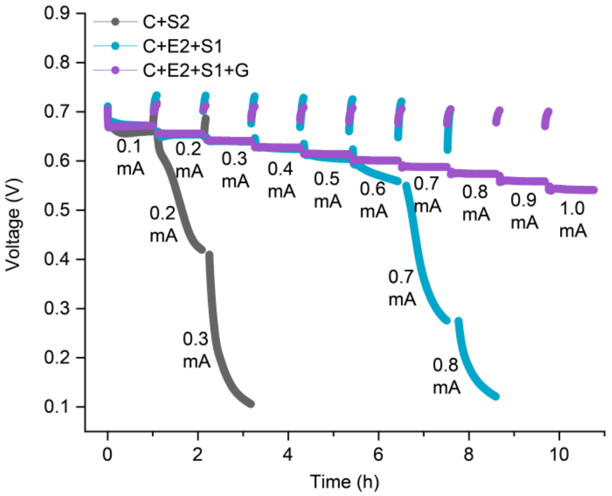

Supplementary Fig. 10. Discharge curves of three synthetic microbial communities. Discharging was conducted at constant currents of 0.1~1.0 mA increased in a step of 0.1 mA. At each current value, the discharging was maintained for 1 h, followed by an open-circuit state for 5 min. Source data are provided as a Source Data file.

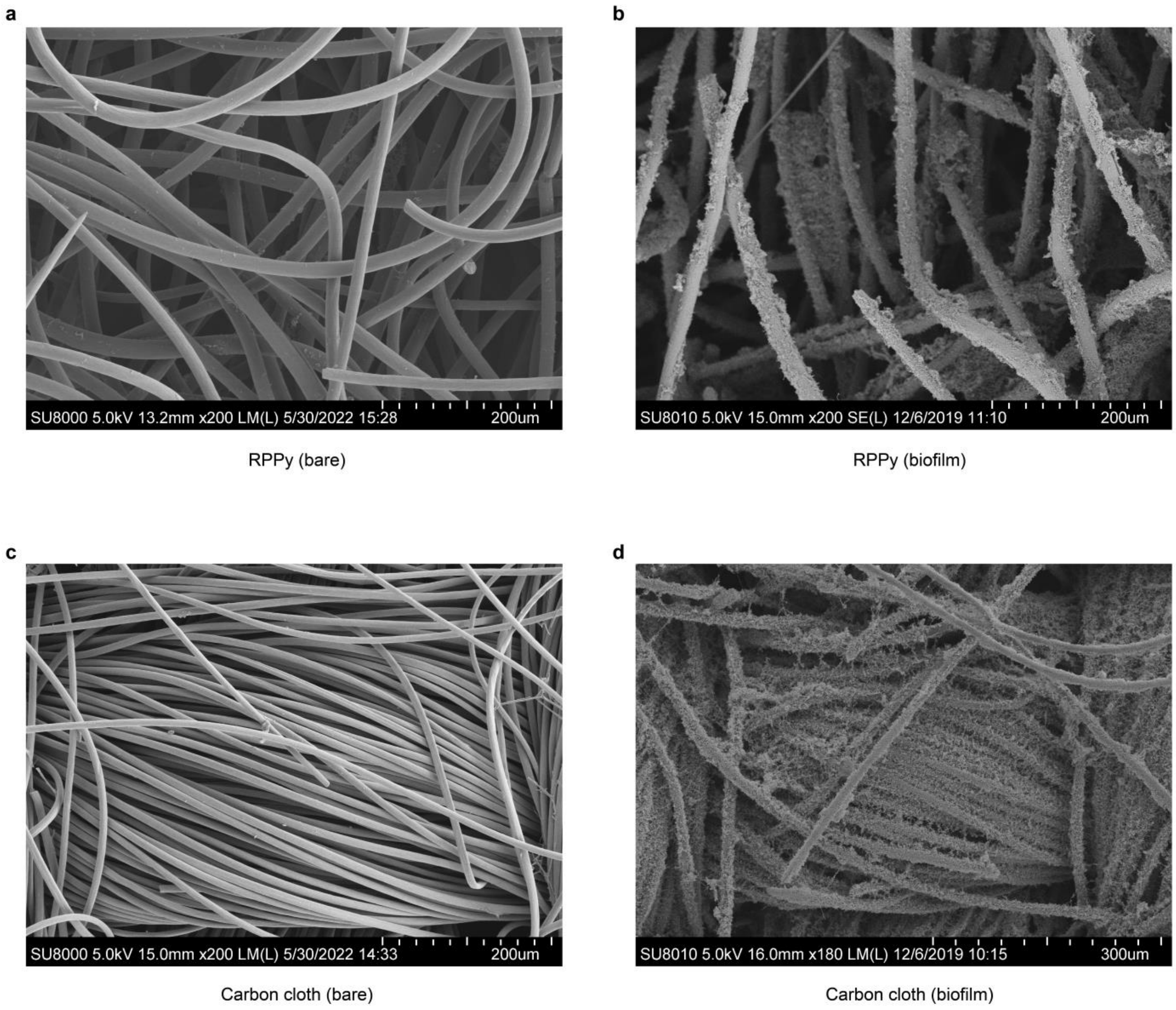

Supplementary Fig. 11. The microscopic morphology of RPPy and carbon cloth. a, SEM image of bare RPPy. **b**, SEM image of RPPy with biofilm. **c**, SEM image of bare carbon cloth. **d**, SEM image of carbon cloth with biofilm. The experiment was repeated three times independently with similar results. Source data are provided as a Source Data file.

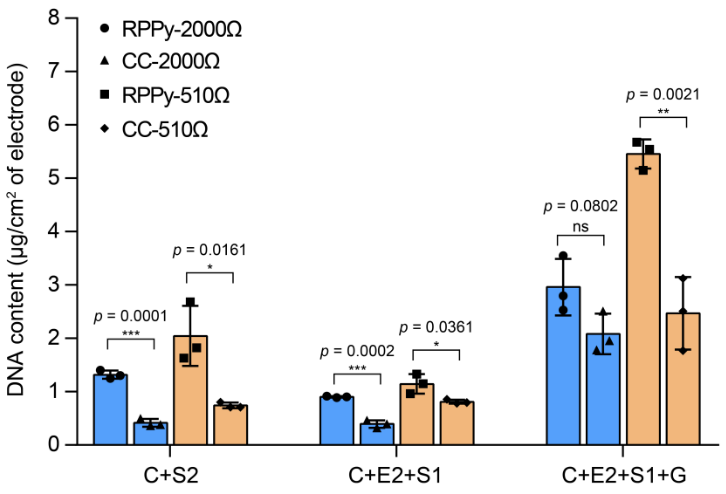

Supplementary Fig. 12. DNA content extracted from different anodic biofilms. Abbreviation: CC: carbon cloth. Data are presented as mean values ± SD from *n* = 3 independent experiments. Two-sided t-test was used to determine the significance (***$p < 0.001$; **$p < 0.01$; *$p < 0.05$; ns: no significant different). Source data are provided as a Source Data file.

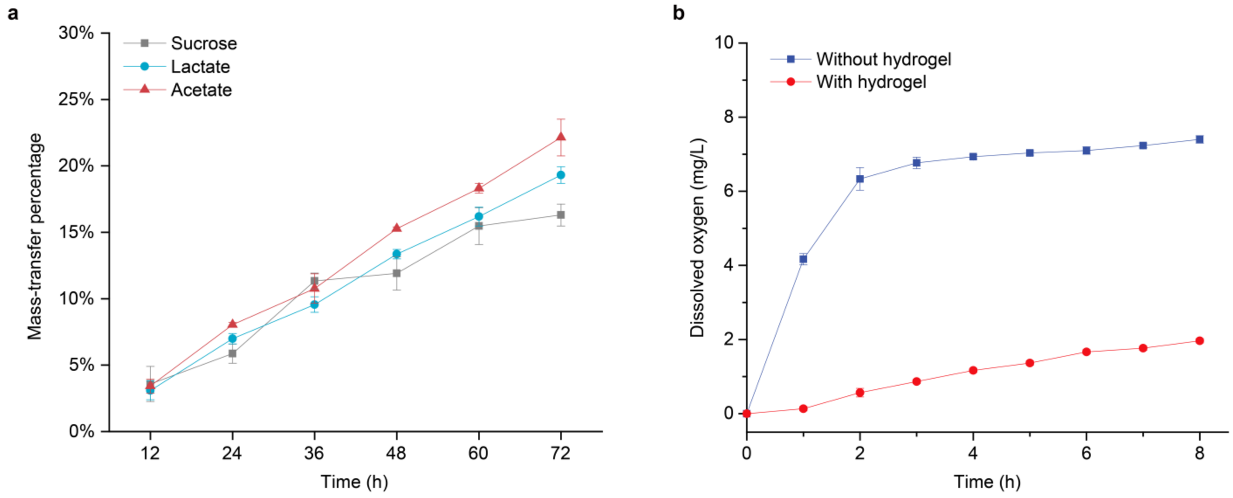

Supplementary Fig. 13. The mass transfer and oxygen permeability of hydrogel. a, The mass transfer of sucrose, lactate and acetate across the hydrogel. The experiments were conducted in a dual-chamber device separated by a hydrogel with 3-mm thickness. Three compounds of approximately 3.0 $g \cdot L^{-1}$ were added to one chamber and stirring magnetically at 500 rpm. The concentrations of sucrose, lactate and acetate in another chamber were measured. **b**, Oxygen permeability across the hydrogel. The experiment was conducted in a dual-chamber device separated by a hydrogel with 3-mm thickness. One chamber was air saturated by stirring magnetically at 500 rpm, and the concentration of dissolved oxygen in another chamber was monitored. Data are presented as mean values ± SD from $n = 3$ independent experiments. Source data are provided as a Source Data file.

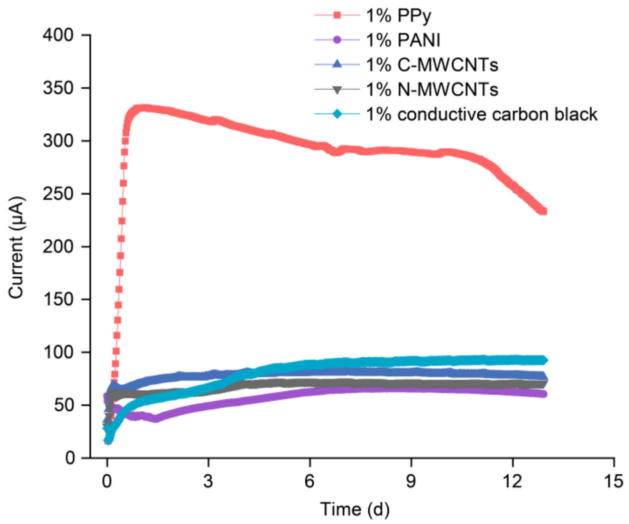

Supplementary Fig. 14. Electricity generated by encapsulated *S. oneidensis* in different conductive hydrogels. The conductive materials included PPy, PANI, C-MWCNTs, N-MWCNTs and conductive carbon black. In this experiment, the aerobic respiration-null mutant of *S. oneidensis* (E3) was used, with 15 mM sodium lactate as electron donor. Source data are provided as a Source Data file.

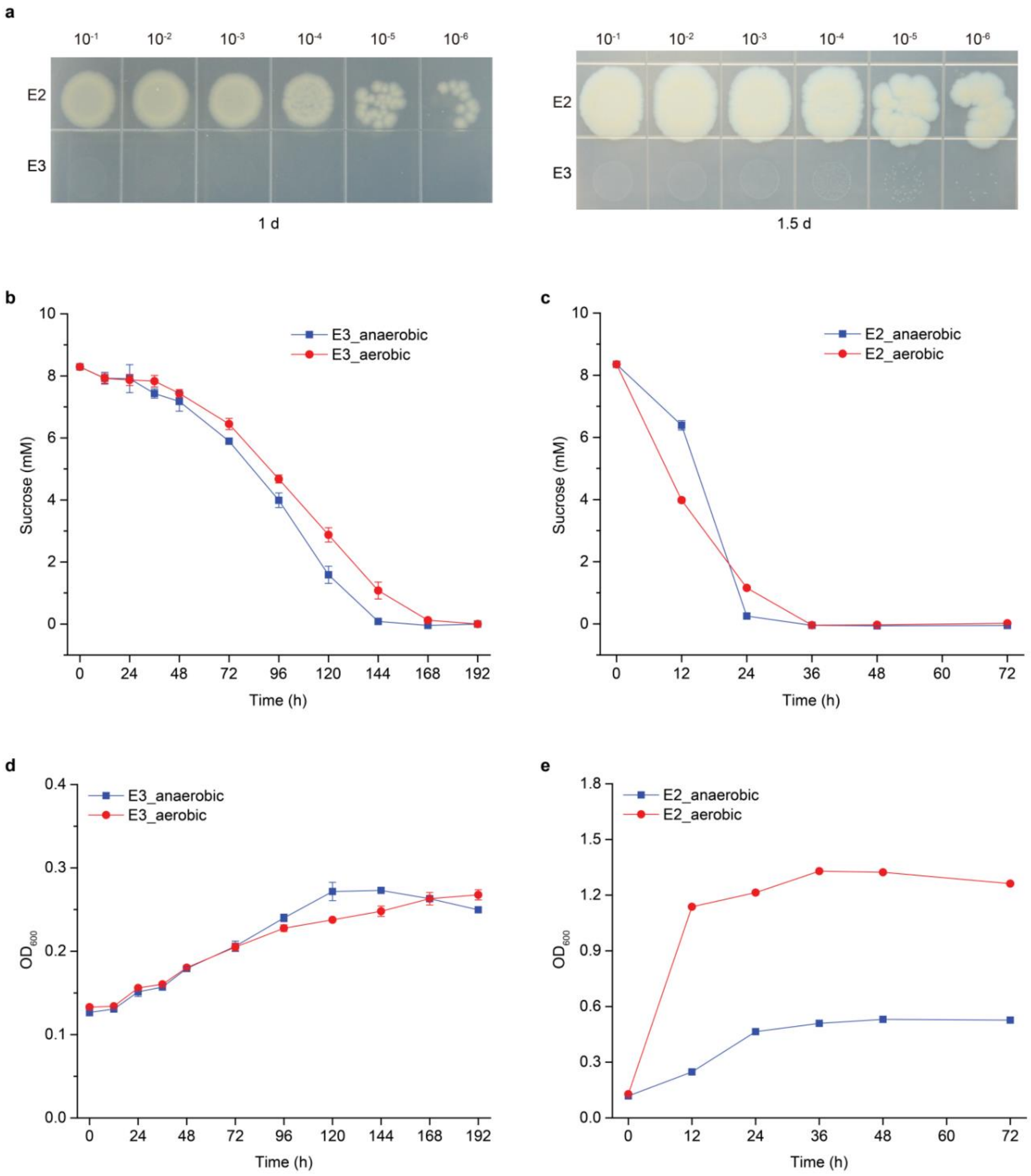

Supplementary Fig. 15. Characterization of the aerobic respiration-null mutant of *E. coli*. a, Drop plate test of strains E2 and E3 under aerobic conditions. **b**,**d** The sucrose consumption (**b**) and growth (**d**) of strain E3 under anaerobic and aerobic conditions. **c**,**e** The sucrose consumption (**c**) and growth (**e**) of strain E2 under anaerobic and aerobic conditions. The fermentation was conducted in MBG11-S medium supplemented with 3 g·L^{-1} sucrose as carbon source. Data are presented as mean values ± SD from n = 3 independent experiments. Source data are provided as a Source Data file.

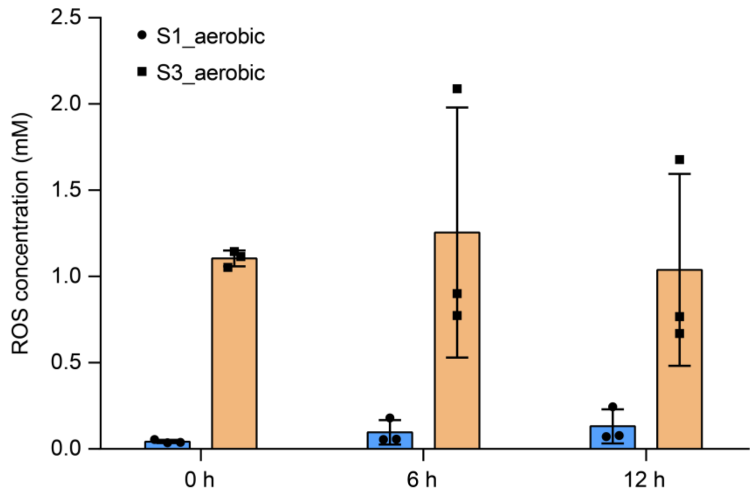

Supplementary Fig. 16. Intracellular ROS concentration of strains S1 and S3 under aerobic conditions. Strain S3 is an aerobic respiration-null mutant. Data are presented as mean values ± SD from $n = 3$ independent experiments. Source data are provided as a Source Data file.

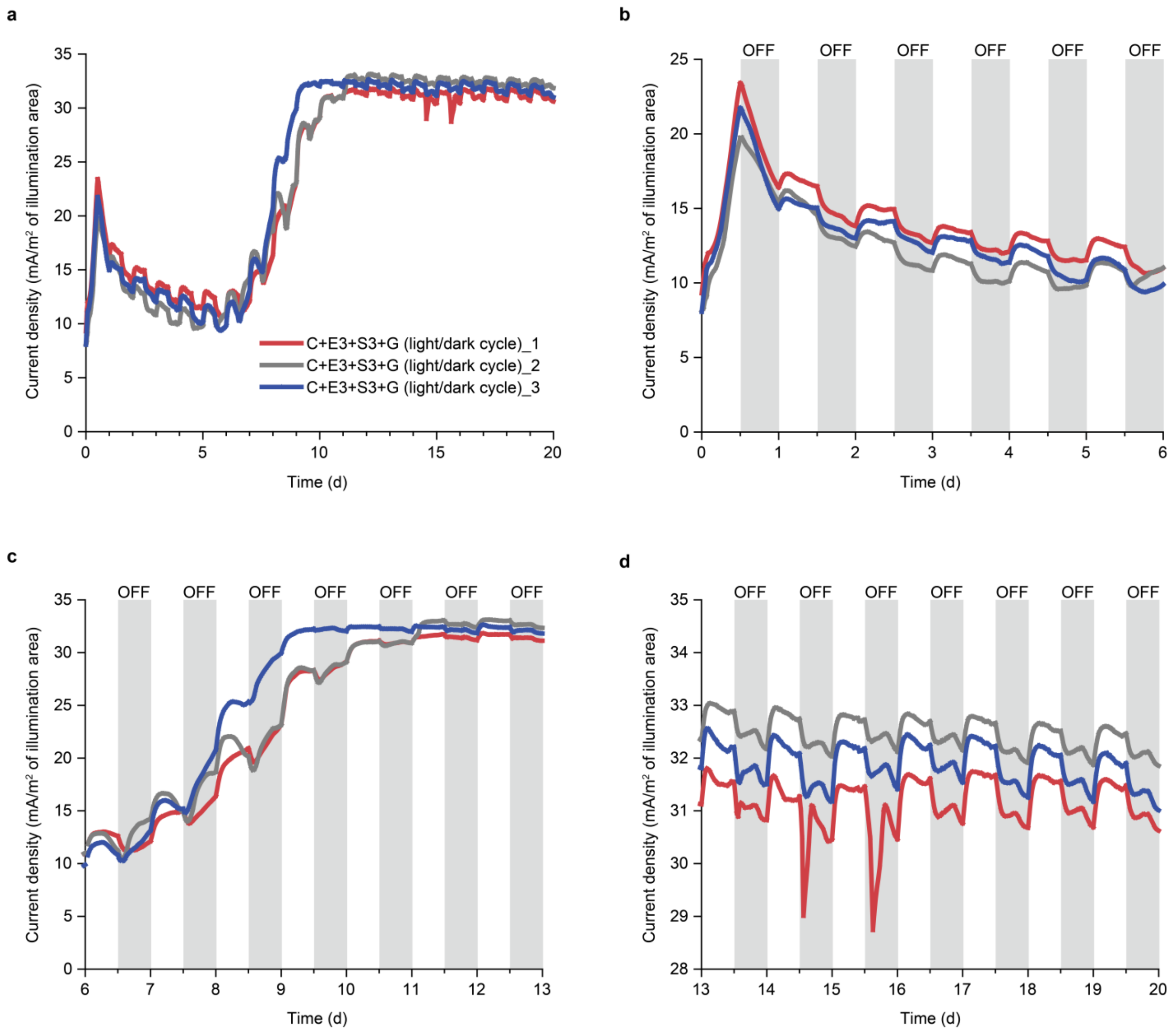

Supplementary Fig. 17. Current density generated by the miniaturized bionic ocean-battery under light/dark cycles (12 h light, 12 h dark). a, The overall current density plots of three independent reactors run for 20 days. **b**, The enlarged view of current density plots at 0~6th days. **c**, The enlarged view of current density plots at 6th~13th days. **d**, The enlarged view of current density plots at 13th~20th days. The dark periods were labeled with gray bars at the panels of **b**, **c** and **d**. The current densities were normalized to the geometrical area of receiving the light. Source data are provided as a Source Data file.

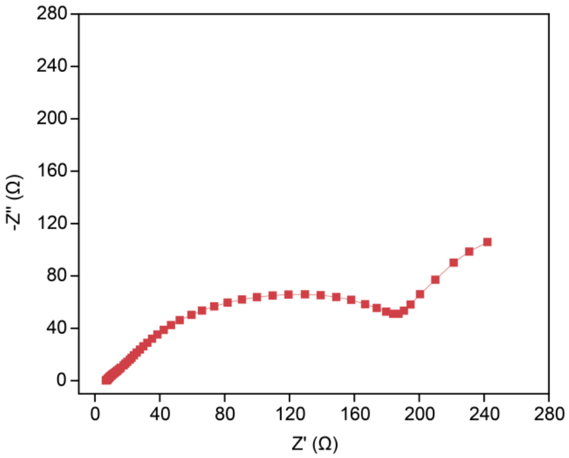

Supplementary Fig. 18. Nyquist plot of the miniaturized bionic ocean-battery. EIS was measured in a frequency range of 100 kHz to 1 mHz at the open circuit potential. Source data are provided as a Source Data file.

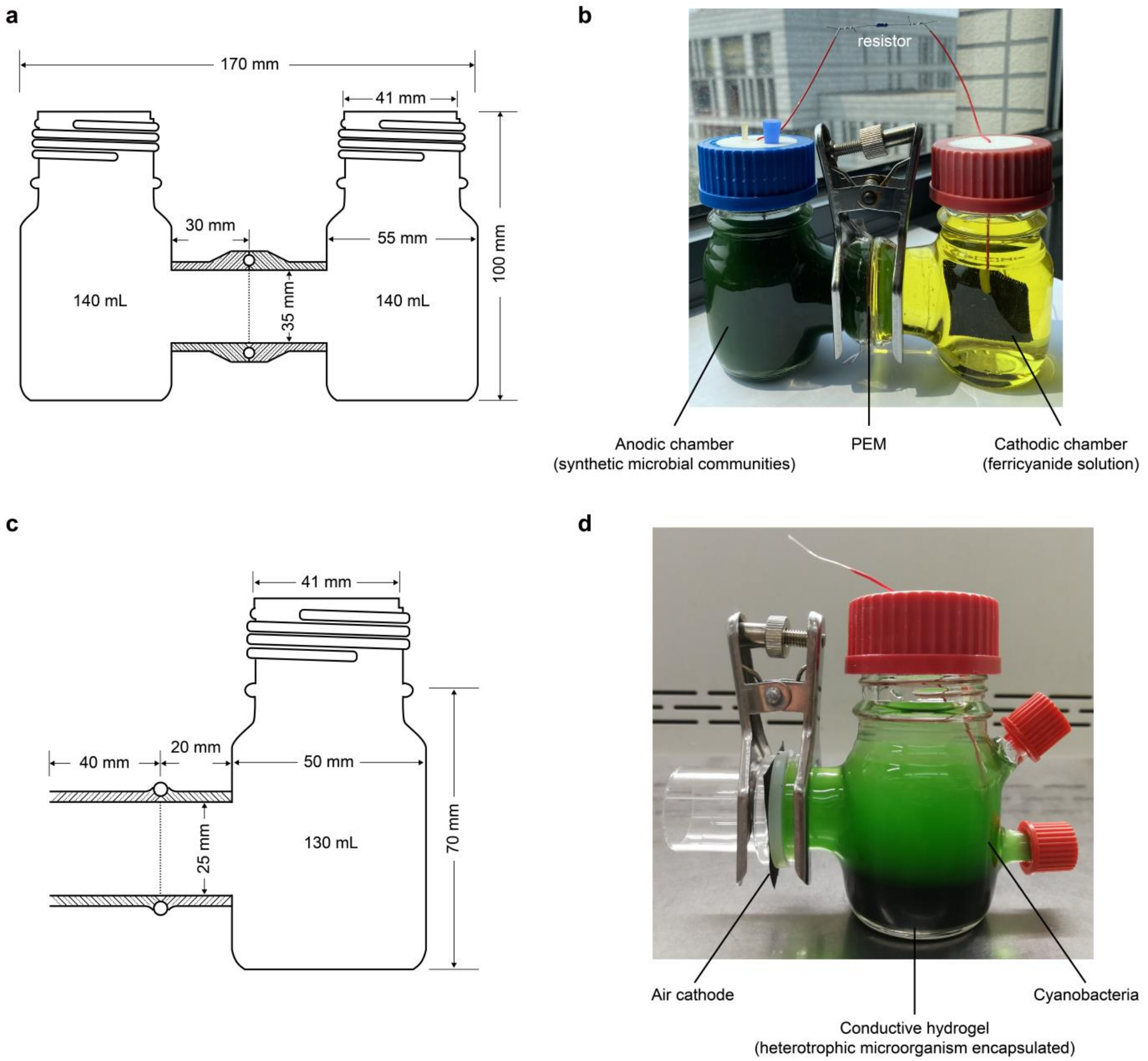

Supplementary Fig. 19. The schematic diagram and photograph of the electrochemical apparatus used in this study. a, The schematic diagram of dual-chamber device, showing the actual geometry and size. **b**, The photograph of dual-chamber device, showing the anodic and cathodic chambers. **c**, The schematic diagram of single-chamber device. **d**, The photograph of single-chamber device, showing the anodic chamber and air cathode. The anodic chamber was composed of conductive hydrogel layer and cyanobacteria layer.

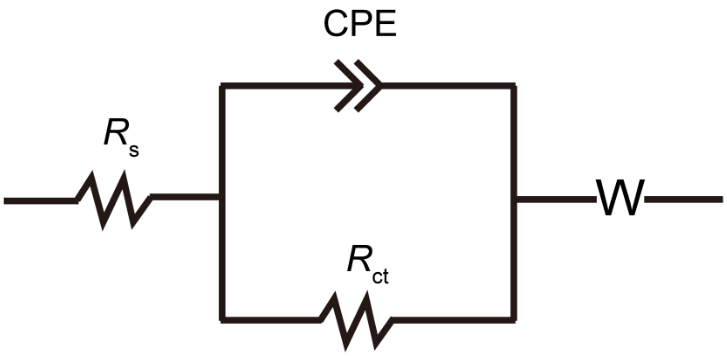

Supplementary Fig. 20. The equivalent electrical circuit model. R_s: solution resistance; R_{ct}: charge transfer resistance of the anode; CPE: constant phase element; W: Warburg diffusion element.

Supplementary Table 1. Strains used in this study.

Abbr.	Strain	Characteristics	Source or reference
**Cyanobacteria**			
C	Syn7942-FL130	A sucrose-secreting strain, derived from *Synechococcus elongatus* PCC 7942; NS1::*Ptrc-sps*-Sp^r NS3::*Plac-cscB*-Cm^r	1
E. coli			
E	*E. coli* Dlac-006	ATCC8739, Δ*pflB*, Δ*frdABCD*, Δ*mgsA*	2
E1	*E. coli* HX030-Suc	Derived from E, *adhE*::$P_{M1\text{-}93}$-*cscB*-P_{rrnB}-*gtfA*, used for D-lactate production from sucrose under anaerobic conditions	This study
E1-1	*E. coli* HX030-Suc-Δ*narG*	Derived from E1; Δ*narG*	This study
E1-2	*E. coli* HX030-Suc-Δ*narG-napA*	Derived from E1; Δ*narG*, Δ*napA*	This study
E2	*E. coli* HX030-Suc-Δ*narG-napA-narZ*	Derived from E1; Δ*narG*, Δ*napA*, Δ*narZ*; suitable for D-lactate production from sucrose in nitrate-containing medium	This study
E3	*E. coli* HX030-Suc-Δ*narG-napA-narZ-cyoABCD-appBC-ygiN-cydAB*	Derived from E2; Δ*cyoABCD*, Δ*appBC*, Δ*ygiN*, Δ*cydAB*; suitable for D-lactate production from sucrose under aerobic conditions	This study
S. oneidensis			
S	*S. oneidensis*-Δ*napA*	Derived from wild-type MR-1, *napA* deletion mutant	3
S1	*S. oneidensis*-Δ*napA* (pBBR1MCS-2)	Derived from S; harboring an empty vector	This study
S1-1	*S. oneidensis*-Δ*napA* (pBBR1-*glk-cscB*)	Derived from S; harboring a *glk-cscB* expression plasmid	This study
S2	*S. oneidensis*-Δ*napA* (pBBR1-*glk-cscAKB*)	Derived from S; harboring a *glk-cscAKB* expression plasmid; suitable for electricity generation directly from sucrose	This study
S3	*S. oneidensis*-Δ*napA-cox-cco-cyd*	Derived from S; terminal oxidases deletion mutant, ΔSO4606-4609, ΔSO2361-2364, ΔSO3284-3286; unable of aerobic respiration	This study
G. sulfurreducens			
G	*G. sulfurreducens* PCA	Wild-type (DSM 12127)	DSMZ

Supplementary Table 2. Plasmids used in this study.

Plasmid	Characteristics	Source or reference
pCas	kan, gam-bet-exo, cas9	4
pTargetF	aadA, guide RNA transcription	4
pTargetF-narG	Derived from pTargetF, narG knockout	This study
pTargetF-napA	Derived from pTargetF, napA knockout	This study
pTargetF-narZ	Derived from pTargetF, narZ knockout	This study
pTargetF-cyoABCD	Derived from pTargetF, cyoABCD knockout	This study
pTargetF-appBC	Derived from pTargetF, appBC knockout	This study
pTargetF-ygiN	Derived from pTargetF, ygiN knockout	This study
pTargetF-cydAB	Derived from pTargetF, cydAB knockout	This study
pBBR1MCS-2	A broad-host-range cloning vector, kan	Lab storage
pBBR1-glk-cscB	Derived from pBBR1MCS-2, glk-cscB	This study
pBBR1-glk-cscAKB	Derived from pBBR1MCS-2, glk-cscAKB	This study
pRE112	A suicide vector, cat	Lab storage
pRE112-SO4606-4609	Derived from pRE112, cox knockout	This study
pRE112-SO2361-2364	Derived from pRE112, cco knockout	This study
pRE112-SO3284-3286	Derived from pRE112, cyd knockout	This study

Abbreviations: cat: chloramphenicol resistance gene; aadA: spectinomycin resistance gene; kan: kanamycin resistance gene; gam-bet-exo: Red recombinase genes; cas9: Cas9 protein coding gene.

Supplementary Table 3. Compositions of BG11 and MBG11-S media used in this study.

Components ($g\cdot L^{-1}$)	BG11	MBG11-S
$Na_2HPO_4\cdot 12H_2O$		17.1
KH_2PO_4		3.0
NaCl		8.766
$MgSO_4$	0.036	0.036
$CaCl_2$	0.027	0.027
$NaNO_3$	1.5	1.5
$K_2HPO_4\cdot 3H_2O$	0.047	
Na_2CO_3	0.02	
Citrate	0.006	0.006
Ammonium ferric citrate	0.006	
EDTA disodium	0.001	0.001
*Trace elements solution (1000×)	1.0 mL	1.0 mL

*(per liter): 2.86 g H_3BO_3, 1.81 g $MnCl_2\cdot 4H_2O$, 0.222g $ZnSO_4\cdot 7H_2O$, 0.079 g $CuSO_4\cdot 5H_2O$, 0.391 g $Na_2MoO_4\cdot 2H_2O$ and 0.04 g $CoCl_2\cdot 6H_2O$.

Supplementary references

1. Duan, Y. K., Luo, Q., Liang, F. Y. & Lu, X. F. Sucrose secreted by the engineered cyanobacterium and its fermentability. *J. Ocean Univ*. **15**, 890-896 (2016).
2. Ma, Y. *et al*. New recombinant *Escherichia coli* useful in production of D-lactic acid, obtained by performing modifications to inhibit *pyruvate formate-lyase B* (*pflB*) gene expression and/or activity of *pflB* gene encoding protein. U.S. Patent WO2015003629-A1; CN104278003-A; EP3020802-A1; US2016145653-A1; EP3020802-A4; US9944957-B2; CN104278003-B; EP3020802-B1 (2018).
3. Gao, H. C. *et al.* Reduction of nitrate in *Shewanella oneidensis* depends on atypical NAP and NRF systems with NapB as a preferred electron transport protein from CymA to NapA. *ISME J.* **3**, 966-976 (2009).
4. Jiang, Y. *et al.* Multigene editing in the *Escherichia coli* genome via the CRISPR-Cas9 system. *Appl. Environ. Microbiol*. **81**, 2506-2514 (2015).
