# Supplementary material for: A miniaturized bionic ocean-battery mimicking the structure of marine microbial ecosystems
Source: Nat Commun. 2022 Sep 24;13:5608. doi: 10.1038/s41467-022-33358-x (PMC9509365; doi:10.1038/s41467-022-33358-x)
Supplement: Supplementary file 3 — Description of Additional Supplementary Files [file 41467_2022_33358_MOESM3_ESM.pdf]

Description of Additional Supplementary Files

File Name: Supplementary Data 1

Description: The performance of previously demonstrated bio-solar cells (by using whole microorganisms).

File Name: Supplementary Data 2

Description: The sequences of primers used in this study.

File Name: Supplementary Data 3

Description: The sequences of sucrose metabolism genes used in this study.
