# Supplementary material for: A miniaturized bionic ocean-battery mimicking the structure of marine microbial ecosystems
Source: Nat Commun. 2022 Sep 24;13:5608. doi: 10.1038/s41467-022-33358-x (PMC9509365; doi:10.1038/s41467-022-33358-x)
Supplement: Supplementary file 7 — Reporting Summary [file 41467_2022_33358_MOESM7_ESM.pdf]

nature portfolio

Corresponding author(s): Xuefeng Lu, Xueli Zhang, Zhiguang Zhu, Yanping Zhang & Yin Li

Last updated by author(s): Sep 6, 2022

Reporting Summary

Nature Portfolio wishes to improve the reproducibility of the work that we publish. This form provides structure for consistency and transparency in reporting. For further information on Nature Portfolio policies, see our Editorial Policies and the Editorial Policy Checklist.

Statistics

For all statistical analyses, confirm that the following items are present in the figure legend, table legend, main text, or Methods section.

n/a	Confirmed	
☐	☒	The exact sample size (*n*) for each experimental group/condition, given as a discrete number and unit of measurement
☐	☒	A statement on whether measurements were taken from distinct samples or whether the same sample was measured repeatedly
☐	☒	The statistical test(s) used AND whether they are one- or two-sided<br>*Only common tests should be described solely by name; describe more complex techniques in the Methods section.*
☒	☐	A description of all covariates tested
☒	☐	A description of any assumptions or corrections, such as tests of normality and adjustment for multiple comparisons
☐	☒	A full description of the statistical parameters including central tendency (e.g. means) or other basic estimates (e.g. regression coefficient) AND variation (e.g. standard deviation) or associated estimates of uncertainty (e.g. confidence intervals)
☐	☒	For null hypothesis testing, the test statistic (e.g. *F*, *t*, *r*) with confidence intervals, effect sizes, degrees of freedom and *P* value noted<br>*Give P values as exact values whenever suitable.*
☒	☐	For Bayesian analysis, information on the choice of priors and Markov chain Monte Carlo settings
☒	☐	For hierarchical and complex designs, identification of the appropriate level for tests and full reporting of outcomes
☒	☐	Estimates of effect sizes (e.g. Cohen's *d*, Pearson's *r*), indicating how they were calculated

Our web collection on statistics for biologists contains articles on many of the points above.

Software and code

Policy information about availability of computer code

Data collection	A Data Acquisition System (FLUKE, WA, USA) was used for voltage data collection. A CHI1030C potentiostat and associated electrohemical software chi1030c (version 16.6.0.0) were used for electrochemical data collection. A CHI660E electrochemical workstation was used for EIS analysis. The Nyquist plots were fitted to an equivalent circuit model using the software ZSimpWin (version 3.60). The constant current discharge was conducted using a CT3001A LANHE battery test system (Wuhan LAND Electronics, China) associated with software LANDMon (version 7.3). A TU-1900 UV–VIS spectrophotometer (Persee, Beijing, China) was used to measure the cell optical density. An Agilent 1260 HPLC system (Agilent Technologies, CA, USA) was used to quantify the concentrations of lactate, acetate, fumarate and succinate.
Data analysis	The raw data of OTUs (Operational Taxonomic Units) were analyzed on the online platform of Majorbio Cloud Platform (www.majorbio.com, version 4.1). Microsoft Excel 2010, Origin 2016 and GraphPad Prism 7 were used for data analysis and graphing.

For manuscripts utilizing custom algorithms or software that are central to the research but not yet described in published literature, software must be made available to editors and reviewers. We strongly encourage code deposition in a community repository (e.g. GitHub). See the Nature Portfolio guidelines for submitting code & software for further information.

Data

Policy information about availability of data

All manuscripts must include a data availability statement. This statement should provide the following information, where applicable:

- Accession codes, unique identifiers, or web links for publicly available datasets
- A description of any restrictions on data availability
- For clinical datasets or third party data, please ensure that the statement adheres to our policy

Data supporting the findings of this work are included within the paper and its Supplementary Information files. Source data are provided with this paper.

Field-specific reporting

Please select the one below that is the best fit for your research. If you are not sure, read the appropriate sections before making your selection.

[x] Life sciences [ ] Behavioural & social sciences [ ] Ecological, evolutionary & environmental sciences

For a reference copy of the document with all sections, see nature.com/documents/nr-reporting-summary-flat.pdf

Life sciences study design

All studies must disclose on these points even when the disclosure is negative.

Sample size	The sample size of N=3 biological replicates was choosen according to the standards of the field (at least three independent biological replicates for statistic analysis) to ensure reproducibility while minimizing cost.
Data exclusions	No data were excluded from analysis.
Replication	All experiments were independently repeated at least three times with good reproducibility.
Randomization	The electrochemical devices and cultivation flasks used in all experiments were randomly placed in illumination/dark incubators.
Blinding	Blinding was not performed because the data collection was recorded by well-controlled instruments. In addition, the data are quantitative and appropriate control experiments were included, so the subjective judgment or interpretation was not involved during data analysis.

Reporting for specific materials, systems and methods

We require information from authors about some types of materials, experimental systems and methods used in many studies. Here, indicate whether each material, system or method listed is relevant to your study. If you are not sure if a list item applies to your research, read the appropriate section before selecting a response.

Materials & experimental systems

n/a	Involved in the study	
[x]	[ ]	Antibodies
[x]	[ ]	Eukaryotic cell lines
[x]	[ ]	Palaeontology and archaeology
[x]	[ ]	Animals and other organisms
[x]	[ ]	Human research participants
[x]	[ ]	Clinical data
[x]	[ ]	Dual use research of concern

Methods

n/a	Involved in the study	
[x]	[ ]	ChIP-seq
[x]	[ ]	Flow cytometry
[x]	[ ]	MRI-based neuroimaging
